# Comparative Evaluation of Child Behavior Checklist-Derived Scales in Children Clinically Referred for Emotional and Behavioral Dysregulation

**DOI:** 10.3389/fpsyt.2016.00146

**Published:** 2016-08-24

**Authors:** Efstathios Papachristou, Kurt Schulz, Jeffrey Newcorn, Anne-Claude V. Bédard, Jeffrey M. Halperin, Sophia Frangou

**Affiliations:** ^1^Department of Primary Care and Population Health, University College London (UCL), London, UK; ^2^Department of Psychiatry, Icahn School of Medicine at Mount Sinai, New York, NY, USA; ^3^Ontario Institute for Studies in Education, University of Toronto, Toronto, ON, Canada; ^4^Department of Psychology, Queens College, New York, NY, USA

**Keywords:** CBCL-MS, CBCL-DP, Externalizing Scale, self-regulation, early detection

## Abstract

**Background:**

We recently developed the Child Behavior Checklist-Mania Scale (CBCL-MS), a novel and short instrument for the assessment of mania-like symptoms in children and adolescents derived from the CBCL item pool and have demonstrated its construct validity and temporal stability in a longitudinal general population sample.

**Objective:**

The aim of this study was to evaluate the construct validity of the 19-item CBCL-MS in a clinical sample and to compare its discriminatory ability to that of the 40-item CBCL-dysregulation profile (CBCL-DP) and the 34-item CBCL-Externalizing Scale.

**Methods:**

The study sample comprised 202 children, aged 7–12 years, diagnosed with DSM-defined attention deficit hyperactivity disorder (ADHD), conduct disorder (CD), oppositional defiant disorder (ODD), and mood and anxiety disorders based on the Diagnostic Interview Schedule for Children. The construct validity of the CBCL-MS was tested by means of a confirmatory factor analysis. Receiver operating characteristics (ROC) curves and logistic regression analyses adjusted for sex and age were used to assess the discriminatory ability relative to that of the CBCL-DP and the CBCL-Externalizing Scale.

**Results:**

The CBCL-MS had excellent construct validity (comparative fit index = 0.97; Tucker–Lewis index = 0.96; root mean square error of approximation = 0.04). Despite similar overall performance across scales, the clinical range scores of the CBCL-DP and the CBCL-Externalizing Scale were associated with higher odds for ODD and CD, while the clinical range scores of the CBCL-MS were associated with higher odds for mood disorders. The concordance rate among the children who scored within the clinical range of each scale was over 90%.

**Conclusion:**

CBCL-MS has good construct validity in general population and clinical samples and is therefore suitable for both clinical practice and research.

## Introduction

The accurate identification of childhood psychopathology represents an important step in formulating early intervention strategies that could improve prognosis. Yet, the task of developing instruments for the assessment of psychiatric conditions in children is challenging because of high comorbidity (1–3) and significant overlap in clinical features (2). Our group has a long-standing interest in childhood emotional and cognitive dysregulation and its relevance to the formal diagnostic categories of attention deficit hyperactivity disorder (ADHD) and bipolar disorder (BD) (4–7). This motivates research into developing instruments for the assessment of childhood behavioral problems that can be easily used in research and clinical care.

We recently developed the Child Behavior Checklist (CBCL)-Mania Scale (MS) (8) that derives from the CBCL. The CBCL is a 118-item parent report instrument that is widely used because of its sound psychometric properties and transcultural validity (9, 10). The CBCL-MS uses only 19 CBCL items (Table S1 in Supplementary Material) chosen to map onto the criteria for mania outlined in DSM-IV and DSM5 (11, 12), while also acknowledging the predictive value and high prevalence of psychotic symptoms during acute mood episodes (13). The psychometric properties of the CBCL-MS were evaluated in a general population sample of 2,230 youth assessed at ages 11, 13, and 16 years (8). The scale was shown to have a four-factor structure corresponding to distraction/disinhibition, psychotic symptoms, increased libido, and sleep problems, which remained stable across all three assessment waves (8). A recent study based on a sample of 474 children and adolescents from Brazil has provided further support for the construct validity of the CBCL-MS in the general population (14).

The objective of this paper is to determine the usefulness of the CBCL-MS in clinical settings and evaluate its performance against two other popular CBCL-derived scales, namely the CBCL-Externalizing Scale (15) and the CBCL-dysregulation profile (CBCL-DP) (16–19).

The CBCL-Externalizing Scale (Table S1 in Supplementary Material) was developed by combining the delinquent behavior and aggressive behavior CBCL subscales (15, 20) and has been used to identify BD (21), oppositional defiant disorder (ODD), and anxiety disorders in clinically enriched samples of youth (22, 23). This scale has been criticized however for being too long, having poor ecological validity (24), and poor concordance with formal psychiatric diagnoses (25, 26). The CBCL-DP (Table S1 in Supplementary Material), also known as the CBCL-pediatric bipolar disorder profile (CBCL-PBD), was developed by combining the CBCL subscales for attention problems, aggressive behavior, and anxiety/depression (16–19, 27, 28). Several studies report a significant and temporally stable association between high CBCL-DP scores in childhood and BD, ADHD, conduct disorder (CD), and ODD (16, 24, 28, 29). However, other reports have not supported these findings (5, 30–33).

The present study examined the psychometric properties of the CBCL-MS in a sample of 202 clinically referred children and compared its discriminative ability for multiple psychiatric diagnoses to that of the CBCL-DP and the CBCL-Externalizing Scale.

## Materials and Methods

### Participants

Details of the study sample are shown in Table 1. The sample comprised 202 children aged 7–12 years (M = 9.04, SD = 1.30; 87.5% male) that had been referred for evaluation to the Mount Sinai Childhood Behavior Disorders Research Team for disruptive behaviors and/or suspected ADHD as part of three separate studies (34–36). Exclusion criteria of the original studies included any medical/neurological condition, psychosis, and pervasive developmental disorders.

**Table 1:** **Baseline characteristics of the sample by study of origin**.

Variable	Study 1 (*n* = 96)	Study 2 (*n* = 92)	Study 3 (*n* = 14)	Total sample (*n* = 202)	*p*-value*
Sex, male *n* (%)	82 (92%)	73 (82%)	13 (93%)	168 (88%)	0.10
Age in years	9.12 (1.30)	8.94 (1.35)	9.20 (1.00)	9.04 (1.30)	0.61
Full Scale IQ	98.18 (15.63)	90.49 (14.41)	93.50 (15.35)	94.31 (15.43)	0.05
CBCL-MS Scale	62.27 (13.31)	64.24 (11.64)	66.00 (18.13)	63.61 (12.93)	0.55
CBCL-DP Scale	65.57 (9.38)	68.93 (10.06)	70.00 (13.24)	67.50 (10.04)	0.09
CBCL-Externalizing Scale	65.00 (12.22)	68.88 (11.08)	63.00 (17.41)	66.70 (12.27)	0.06
Mood disorders, *n* (%)	13 (16%)	9 (10%)	1 (7%)	23 (13%)	0.47
CD, *n* (%)	15 (18%)	33 (38%)	4 (29%)	52 (29%)	0.02
ODD, *n* (%)	51 (62%)	68 (79%)	8 (57%)	127 (70%)	0.03
Anxiety disorders, *n* (%)	26 (32%)	29 (34%)	1 (7%)	56 (31%)	0.13
ADHD, *n* (%)	64 (74%)	81 (94%)	12 (86%)	154 (85%)	0.002

*p-Value of respective Chi-square test or one-way ANOVA.

*Proportions (%) reported are relative to the total number of participants with complete data for each respective variable*.

*Continuous variables shown as Mean (SD), unless otherwise specified*.

*CBCL, Child Behavior Checklist; MS, Mania Scale; DP, dysregulation profile; CD, conduct disorder; ODD, oppositional defiant disorder; ADHD, attention deficit hyperactivity disorder*.

Formal diagnoses were based on parental reports using the Diagnostic Interview Schedule for Children (DISC) version 2.1 in 111 children (37) and version 2.3 in 91 children (38). The diagnoses considered included mood disorders, mainly major depressive disorder and dysthymia, ADHD, CD, ODD, and anxiety disorders. In total, 23 children were diagnosed with a mood disorder (13%), 56 with an anxiety disorder (31%), 154 with ADHD (85%), 52 with CD (29%), and 127 with ODD (70%). Nineteen children did not receive any diagnosis (10.4%), while 133 (73%) were comorbid for two or more disorders. The range of the full scale IQ of the analyses sample was 60–139, with 7% (*n* = 13) of children having IQ scores <70.

Parents of all children completed the CBCL (20) during clinic visits. Cumulative scores ≥210 on the attention problems, aggressive behavior, and anxious/depressed CBCL scales upon standardization (*T* scores) were considered significantly elevated scores for the CBCL-DP (27, 28). Total standardized *T* scores ≥70 (2 SDs above the mean) were considered significantly elevated scores for the CBCL-MS and the CBCL-Externalizing Scale. Full scale, verbal, and performance IQ were assessed using the Wechsler Intelligence Scale for Children-Revised (WISC-R) in 96 children and the WISC-III in 106 children (39, 40). The differences observed in the cognitive abilities of children across samples were fully accounted for by the shift from WISC-R to WISC-III (Table 1).

### Statistical Analysis

We performed confirmatory factor analyses (CFA) to examine whether the four-factor structure of the CBCL-MS previously described in a general population sample could be validated in referred children. Goodness of fit was determined using four indices, the comparative fit index (CFI) (cutoff values above 0.95 indicate good fit), the Tucker–Lewis index (TLI) (cutoff values above 0.95 indicate good fit), the root mean square error of approximation (RMSEA) (cutoff values below 0.06 indicate good fit), and the relative (also called normed) Chi-Square (χ^2^ divided by the degrees of freedom of the model; cutoff values below 2 indicate good fit) (41, 42). The fit of the CFA model was estimated using the weighted least squares, mean, and variance (WLSMV) estimator. Minor model modifications were performed using Mplus’ modification indices by allowing correlations between the unique variances of some individual items within the same factors. Such model modifications do not alter the substantive conclusions regarding the factor structure yet improve model fit by increasing the proportion of the variance explained (43). We bootstrapped the CFA model to obtain more reliable estimates for the 95% confidence interval (CI) of the factor loadings of individual items on their respective factors (44).

The discriminative ability of the CBCL-MS, CBCL-DP, and the CBCL-Externalizing Scale for DSM-based diagnoses was assessed using receiver operating characteristic (ROC) curves. Areas under the curve (AUC) were compared across scales for each DSM diagnosis using the Stata command *roccomp*. The total scores of scales were used as continuous variables for the ROC curves; the CBCL-DP scores were divided by three to ensure identical range of scores for the three scales. Differences in total mean scores of the scales were compared between cases with ADHD, CD, ODD, anxiety disorders, or mood disorders and non-cases with a series of *t*-tests. Upon identification of children with significantly elevated scores on CBCL-MS, CBCL-DP, or CBCL-Externalizing Scale, we ran a series of logistic regression models to assess age-, sex-, and sample-adjusted odds ratios (ORs) and 95% CIs with respect to multiple diagnostic outcomes. Non-cases were used as the reference category for each respective regression model. Additional ROC–AUC and *t*-tests were performed to examine the discriminative ability of the items that are unique to the CBCL-MS and those that are shared between the CBCL-MS and the other two scales. Analyses were performed using Stata/SE 14.0 (StataCorp, College Station, TX, USA) and Mplus v.6 (www.statmodel.com).

## Results

### Factor Structure and Internal Consistency of the CBCL-MS

The results of the CFA are shown in Table 2. Standardized factor loadings yielded a four-factor structure representing distraction/disinhibition, increased libido, sleep problems, and psychotic symptoms. The four-factor structure of the CBCL-MS showed excellent fit to the data as all four fit indices were well within the recommended cutoffs (CFI = 0.97; TLI = 0.96; RMSEA = 0.04; and relative Chi-square = 1.32). The internal consistency of the CBCL-MS was also high (Cronbach’s alpha = 0.83). For the individual factors, internal consistency rates were 0.78 for distraction/disinhibition; 0.68 for psychotic symptoms; 0.83 for increased libido (upon removal of item 96 “thinks of sex too much” as the results suggested significant increase of internal consistency); and 0.69 for troubled sleep.

**Table 2:** **Factor loadings, SE, and bootstrapped 95% confidence interval of the CBCL-MS items**.

Factors	Items	Standardized factor loadings	SE	95% bootstrap CI
Distraction/disinhibition	Gets in many fights	0.58	0.07	0.45–0.71
	Sudden changes in mood or feelings	0.72	0.06	0.60–0.81
	Showing off or clowning	0.58	0.07	0.42–0.70
	Teases a lot	0.75	0.06	0.63–0.84
	Talks too much	0.57	0.07	0.43–0.69
	Unusually loud	0.67	0.07	0.51–0.79
	Cannot sit still, restless, or hyperactive	0.60	0.08	0.41–0.74
	Impulsive or acts without thinking	0.75	0.06	0.64–0.85
Psychotic symptoms	Feels others are out to get him/her	0.80	0.07	0.66–0.92
	Strange ideas	0.74	0.09	0.54–0.88
	Suspicious	0.82	0.05	0.71–0.91
	Hears sounds or voices that are not there	0.56	0.12	0.30–0.79
	Sees things that are not there	0.40	0.16	0.08–0.67
Increased libido	Thinks about sex too much	0.61	0.14	0.30–0.83
	Plays with own sex parts too much	0.68	0.13	0.35–0.98
	Plays with own sex parts in public	0.60	0.19	0.25–1.00
Troubled sleep	Trouble sleeping	0.86	0.12	0.65–1.11
	Sleeps less than most kids	0.79	0.10	0.58–0.99

### Discriminative Ability of the Three Scales

**Figure 1:**
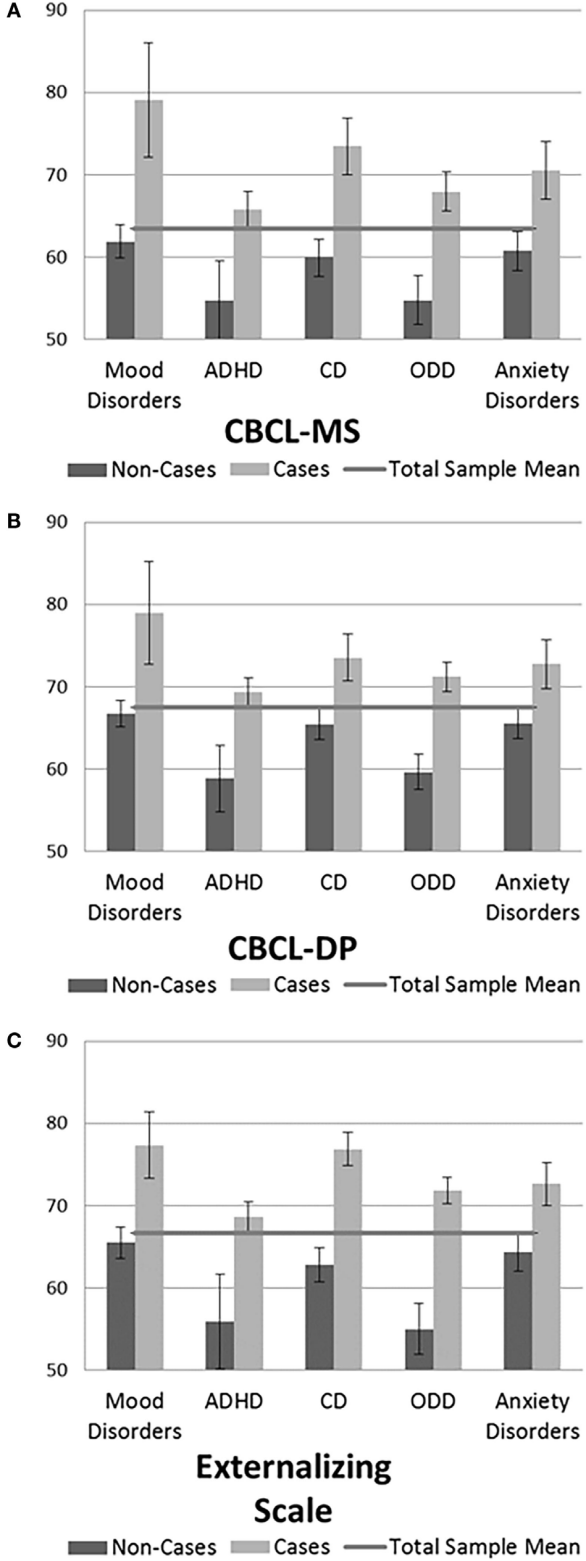
**(A–C)** Mean (±2*SE) CBCL-Mania Scale, CBCL-dysregulation profile (CBCL-DP), and CBCL-Externalizing Scale *T*-scores across diagnostic categories.

Figures 1A–C illustrate the mean score differences of each scale across diagnostic categories. A series of independent samples’ *t*-tests showed that the mean differences in the scores of the CBCL-MS, the CBCL-DP, and the CBCL-Externalizing Scale between cases and non-cases were all statistically significant (all *p* values <0.01).

The results of the ROC curve analyses for the CBCL-MS, the CBCL-DP, and the CBCL-Externalizing Scale with respect to psychiatric outcomes are shown in Table 3. For mood disorders, the highest AUC was observed for the CBCL-MS (AUC = 0.82; 95% CI 0.71–0.93), followed by the CBCL-Externalizing Scale (AUC = 0.79; 95% CI 0.68–0.89) and then the CBCL-DP (AUC = 0.78; 95% CI 0.64–0.92); pair-wise comparisons showed that these AUC values were not significantly different (*p* = 0.30). The CBCL-MS achieved sensitivity rates of 70% and specificity rates of 71%. CBCL-Externalizing Scale achieved sensitivity and specificity rates of 80 and 59%, respectively, and the respective values for the CBCL-DP were 64 and 67%. Comparisons of the extracted AUC values suggest that the three scales have similar discriminative power for anxiety disorders and ADHD (*p* values >0.05). However, the CBCL-Externalizing Scale appears to have increased discriminative power for CD (*p* < 0.001) and ODD (*p* = 0.02).

**Table 3:** **Discriminative abilities of the CBCL-MS, the CBCL-DP, and the CBCL-Externalizing Scale total and associated factor scores for various psychiatric disorders**.

	Area under the curve (95% CI)				
	Mood disorders	ADHD	Anxiety disorders	CD	ODD
*CBCL-MS*	0.82 (0.71–0.93)	0.74 (0.62–0.86)	0.72 (0.64–0.80)	0.80 (0.73–0.88)	0.80 (0.73–0.88)
Distraction/disinhibition	0.79 (0.69–0.89)	0.76 (0.64–0.88)	0.71 (0.63–0.79)	0.82 (0.75–0.88)	0.80 (0.73–0.88)
Psychotic symptoms	0.77 (0.65–0.89)	0.56 (0.46–0.67)	0.62 (0.53–0.71)	0.66 (0.57–0.75)	0.69 (0.62–0.77)
Increased libido	0.61 (0.50–0.71)	0.61 (0.56–0.66)	0.53 (0.47–0.60)	0.55 (0.48–0.62)	0.57 (0.51–0.63)
Sleep problems	0.55 (0.46–0.65)	0.54 (0.48–0.60)	0.54 (0.48–0.60)	0.56 (0.50–0.63)	0.58 (0.54–0.62)
*CBCL-DP*	0.78 (0.64–0.92)	0.80 (0.67–0.92)	0.70 (0.61–0.79)	0.73 (0.64–0.81)	0.84 (0.77–0.91)
Attention problems	0.72 (0.61–0.84)	0.83 (0.73–0.93)	0.61 (0.52–0.70)	0.59 (0.50–0.68)	0.72 (0.63–0.81)
Aggressive behavior	0.75 (0.64–0.87)	0.75 (0.64–0.86)	0.68 (0.60–0.77)	0.82 (0.76–0.89)	0.87 (0.82–0.93)
Anxious/depressed	0.84 (0.72–0.96)	0.69 (0.57–0.81)	0.73 (0.65–0.82)	0.64 (0.54–0.73)	0.78 (0.70–0.86)
*CBCL-Externalizing Scale*	0.79 (0.68–0.89)	0.75 (0.64–0.87)	0.69 (0.61–0.77)	0.85 (0.80–0.91)	0.88 (0.82–0.93)
Delinquent behavior	0.70 (0.58–0.83)	0.67 (0.55–0.79)	0.64 (0.55–0.72)	0.83 (0.76–0.89)	0.81 (0.74–0.87)
*p-Value of ΔAUC*^a^	0.30	0.30	0.38	<0.001	0.02
				Externalizing > CBCL-MS**	Externalizing > CBCL-MS*
				Externalizing > CBCL-DP**	

*^a^Difference of ROC–AUC obtained by the CBCL-MS, CBCL-DP, and CBCL-Externalizing Scale total scores*.

*p < 0.05.

**p < 0.01.

*The Aggressive Behavior scale is shared between the CBCL-DP and the Externalizing Scale*.

*CBCL, Child Behavior Checklist; MS, Mania Scale; DP, dysregulation profile; ADHD, attention deficit hyperactivity disorder; CD, conduct disorder; ODD, oppositional defiant disorder*.

### Associations between the Clinical Range of the Three Scales and Multiple Psychiatric Disorders

A series of logistic regressions was performed to obtain sex, age, and sample of origin adjusted ORs (95% CI) for children with elevated scores on the three scales with respect to multiple psychiatric diagnoses. Results are summarized in Table 4. Overall, 57 (34%) children were found to have CBCL-MS scores ≥70, 53 (34%) had CBCL-DP scores ≥210, and 85 (45%) had scores ≥70 on the CBCL-Externalizing Scale. The Goodman and Kruskal’s gamma for the distributions between dichotomized scores on the CBCL-MS and both scores on the CBCL-DP (γ = 0.90, *p* < 0.001) and the CBCL-Externalizing Scale (γ = 0.94, *p* < 0.001), and also between scores on the CBCL-DP and the CBCL-Externalizing Scale (γ = 0.93, *p* < 0.001), suggested that there was great overlap in the children identified as having elevated scores by all three scales. Mean total scores ≥70 on the CBCL-MS were associated with a sevenfold increase in the risk of being diagnosed with a mood disorder (OR = 7.1, 95% CI 2.2–22.7) or CD (OR = 7.2; 95% CI 3.2–15.9), a sixfold increase for ODD (OR = 6.4, 95% CI 2.4–17.3), and a fourfold increase for anxiety disorders (OR = 4.1, 95% CI 1.9–8.6). However, scoring high only on the distraction/disinhibition (OR = 3.3, 95% CI 1.2–9.0) scale of the CBCL-MS, but not on the total CBCL-MS score (OR = 2.3, 95% CI 0.8–6.7), was significantly associated with ADHD.

**Table 4:** **Cross-sectional associations between the CBCL-MS, CBCL-DP, CBCL-Externalizing Scale, and associated factors with multiple diagnostic outcomes**.

	Mood disorders (*n* = 23; 13%)	ADHD (*n* = 154; 85%)	CD (*n* = 52; 29%)	ODD (*n* = 127; 70%)	Anxiety disorders (*n* = 56; 31%)
*CBCL-MS*					
Score ≥70 (Raw score ≥14), *n* (%)	12 (71%)	47 (38%)	29 (66%)	46 (44%)	26 (54%)
OR (95% CI)	7.06 (2.19–22.69)**	2.26 (0.77–6.66)	7.15 (3.21–15.94)**	6.40 (2.36–17.34)**	4.06 (1.92–8.61)**
Distraction/disinhibition					
Score ≥70 (Raw score ≥10), n (%)	16 (80%)	67 (48%)	39 (78%)	64 (55%)	36 (68%)
OR (95% CI)	6.46 (2.00–20.84)**	3.30 (1.21–9.01)*	8.76 (3.91–19.64)**	5.82 (2.54–13.34)**	4.90 (2.36–10.15)**
Psychotic symptoms					
Score ≥70 (Raw score ≥3), *n* (%)	12 (60%)	30 (21%)	18 (38%)	31 (27%)	17 (33%)
OR (95% CI)	8.45 (3.00–23.84)**	1.22 (0.41–3.64)	4.07 (1.79–9.24)**	4.97 (1.61–15.37)**	3.08 (1.38–6.87)**
Increased libido					
Score ≥70 (Raw score ≥3), *n* (%)	9 (41%)	39 (26%)	15 (29%)	33 (26%)	15 (27%)
OR (95% CI)	3.21 (1.22–8.46)*	9.20 (1.19–71.66)*	1.78 (0.81–3.87)	2.72 (1.07–6.92)*	1.64 (0.77–3.52)
Sleep problems					
Score ≥70 (Raw score ≥1), *n* (%)	5 (24%)	24 (16%)	12 (24%)	24 (19%)	11 (20%)
OR (95% CI)	2.21 (0.71–6.93)	2.51 (0.54–11.69)	2.93 (1.18–7.29)*	7.73 (1.65–36.12)*	1.94 (0.79–4.75)
*CBCL-DP*					
Score ≥210, *n* (%)	9 (64%)	50 (40%)	25 (56%)	50 (48%)	26 (54%)
OR (95% CI)	3.56 (1.03–12.24)*	3.97 (1.05–15.07)*	3.55 (1.60–7.91)**	10.71 (3.05–37.60)**	3.21 (1.51–6.84)**
*CBCL-Externalizing Scale*					
Score >70 *n* (%)	16 (80%)	74 (49%)	44 (86%)	75 (60%)	34 (63%)
OR (95% CI)	5.90 (1.81–19.17)**	2.72 (0.98–7.56)	15.84 (6.32–39.74)**	13.13 (4.80–35.87)**	2.85 (1.42–5.72)**

*Odds ratios adjusted for sex, age, and origin sample*.

*MS, Mania Scale; DP, dysregulation profile; ADHD, attention deficit hyperactivity disorder; CD, conduct disorder; ODD, oppositional defiant disorder*.

*Reference is the control group for each diagnostic category (non-cases)*.

*p < 0.05.

**p < 0.001.

Total scores ≥70 on the CBCL-Externalizing Scale were associated with diagnoses of mood disorder (OR = 5.9, 95% CI 1.8–19.2), anxiety disorder (OR = 2.9, 95% CI 1.4–5.7), CD (OR = 15.8, 95% CI 6.3–39.7), or ODD (OR = 13.1, 95% CI 4.8–35.9), but not ADHD (OR = 2.7, 95% CI 1.0–7.6). Finally, CBCL-DP scores ≥210 were strongly associated with ODD (OR = 10.7, 95% CI 3.1–37.6). High scores on the CBCL-DP were weaker, yet also significantly associated with mood disorders (OR = 3.6, 95% CI 1.0–12.2), anxiety disorders (OR = 3.2, 95% CI 1.5–6.8), ADHD (OR = 4.0, 95% CI 1.1–15.1), and CD (OR = 3.6, 95% CI 1.6–7.9).

### Discriminative Ability of Items Unique to the CBCL-MS

Ten CBCL-MS items were not shared with the other two scales (Table S1 in Supplementary Material). The mean scores of these 10 items were significantly higher for children with a mood disorder (M = 7.76, SD = 3.98) in comparison to those with a diagnosis of ADHD, CD, ODD, or anxiety disorder (M = 4.05, SD = 2.71), *p* < 0.001. Additional sensitivity analyses for the ROC–AUC values of individual items that are unique to the CBCL-MS showed that items 40 (“hears sound or voices that are not there”), 59 (“plays with own sex parts in public”), and 76 (“sleeps less than most kids”) could not individually discriminate between mood and non-mood disorders. For the remaining items, the individual ROC–AUC values ranged from 0.60 (95% CI 0.51–0.70) for item 70 (“sees things that are not there”) to 0.71 (95% CI 0.59–0.82) for item 34 (“feels others are out get him/her”).

## Discussion

The results of this study suggest that CBCL-derived scales have comparable overall performance in the assessment of children referred for problems with behavioral and emotional self-regulation. Within this context, the CBCL-MS may have some advantages over the other scales. First, the factor structure of the CBCL-MS appears robust as it is identical in clinically referred and general population samples of children (8). Second, although its discriminative ability for mood disorders was comparable to that observed for the CBCL-DP and the CBCL-Externalizing Scale, the sensitivity/specificity achieved (70/71%) was more balanced relative to those of the other scales. Third, having just 19 items, the CBCL-MS is a short and versatile instrument in comparison to both the CBCL-DP (40 items) and the CBCL-Externalizing Scale (34 items) while retaining high internal consistency (84%).

The CBCL-MS showed the strongest association with mood disorders in terms of the OR obtained after adjustments for relevant covariates compared to the CBCL-DP and the CBCL-Externalizing Scale. This may reflect the fact that the items comprising the CBCL-MS were selected to map onto DSM diagnostic criteria for mania (8). In contrast, the CBCL-DP was only weakly associated with mood disorders, which conforms with findings suggesting that the CBCL-DP is not necessarily related to BD, but rather to CD, ODD, and ADHD (32). Moreover, the CBCL-MS is the only scale of the three to take into account extended (psychotic) symptoms of BD in addition to core symptoms. A study in a representative community sample of adolescents and young adults has demonstrated that up to 27% of youth with mood or anxiety disorders also displayed one or more psychotic symptoms (45). Accordingly, psychotic symptoms were associated with a 12-fold increased risk of having received a diagnosis of a mood disorder in this sample; moreover, the highest ROC–AUC of the individual items unique to the CBCL-MS was obtained for one of the items loading on the psychotic symptoms factor (“feels others are out to get him/her”).

The ROC–AUCs observed for the three scales were significant for multiple diagnostic outcomes and ranged from 72 to 82% for the CBCL-MS, 70 to 84% for the CBCL-DP, and 69 to 88% for the CBCL-Externalizing Scale. This was expected since the three scales have several items in common (Table S1 in Supplementary Material). Notably, however, the addition of items from the Thought Problems CBCL-subscale may have contributed to the improved discriminability of the CBCL-MS for mood disorders.

It is also worth noting that while the CBCL-MS and the CBCL-DP were initially developed to screen for pediatric BD, they appear to have high discriminatory power for several other diagnostic entities. It is our view that this reflects the fact that these instruments tap into dimensions of poor self-regulation that are relevant to multiple psychiatric diagnoses. Affective dysregulation and attentional dysfunction are also common in children with BD, ADHD, CD, ODD, and disruptive mood dysregulation disorder (DMDD) (2, 46, 47). This is consistent with the high levels of comorbidity observed (1–3, 48, 49). Still, the absence of specificity for DSM-diagnoses does not diminish the importance of the instruments evaluated here, as they can contribute toward the identification of pluripotential early high-risk phenotypes (50). Screening in general population samples could also serve as a two-step approach to identify children who would benefit from clinical referral and detailed clinical assessments (51).

### Strengths and Limitations

This is the first study to compare the commonly examined CBCL-DP and CBCL-Externalizing Scale directly with the newly developed CBCL-MS in a sample of clinically referred children with various psychiatric outcomes. Clinical diagnoses were ascertained using established structured instruments, which are well-validated in clinical samples of this age group (38, 52). Moreover, clinical diagnoses and CBCL assessments were captured almost contemporaneously; they are, therefore, free from recall or attribution biases. The number of patients with BD within the analysis sample was small (*n* = 11). The CFA conducted to assess the construct validity of the CBCL-MS was performed for the whole sample so that the extracted factors reflect the underlying (latent) trait variance of the entire sample of children referred for emotional and behavioral dysregulation. The item pool of the CBCL-MS covers behavioral manifestations of mania, such as inattention/distractibility, hyperactivity, loudness, over-talkativeness, and disrupted sleep that are shared across different diagnostic entities. It would indeed be interesting in a future study with larger numbers of cases with different diagnoses to replicate the findings of this study by assessing the measurement invariance of the factors across diagnostic groups. Still, we believe that for the purpose of this study, the sample size was adequate as it was within the recommended limits of several rules of thumb reviewed in Velicer and Fava (53), e.g., 10 cases for each item in the instrument being used. Most importantly, the estimated SEs of the factor loadings were almost identical to the bootstrap-corrected confidence intervals, suggesting a stable factor structure for the CBCL-MS in our sample.

There are two inherent limitations of the CBCL, which are inevitably reflected in all CBCL-based screening scales. First, behavioral ratings alone are unlikely to yield high levels of accuracy in case identification for any mental disorder. Second, the CBCL items do not fully capture episodicity, which is considered a salient predictor of conversion to BD particularly in young people (29, 54). It is therefore possible that adding more refined information about episodicity may further enhance the predictive value of the scales considered. Moreover, the results of this study are based on a relatively small sample, and it is therefore possible that some of the analyses might have been underpowered. Although we demonstrate the stability of the factor structure of the CBCL-MS in a new independent sample, the longitudinal stability of these factors in clinical samples has yet to be determined.

## Conclusion

The three CBCL-derived scales considered here performed similarly, and the brevity and robust factor structure of the CBCL-MS are distinct benefits. All three scales seem to identify children with difficulties in self-regulation that render them vulnerable to adverse psychiatric outcomes.

## Ethics Statement

Ethical approval was provided by the Institutional Review Board of the Icahn School of Medicine at Mount Sinai. Written consent was sought from parents, and assent was sought from children participating in the original studies based on detailed information about the study protocols.

## Author Contributions

EP conducted the data analyses and contributed to writing the manuscript. KS and A-CB collected the data and contributed to data analysis and manuscript writing. JN, JH, and SF contributed to study conception, data analysis, and manuscript writing.

## Conflict of Interest Statement

The authors declare that the research was conducted in the absence of any commercial or financial relationships that could be construed as a potential conflict of interest.
