# Supplementary material for: Comparative Evaluation of Child Behavior Checklist-Derived Scales in Children Clinically Referred for Emotional and Behavioral Dysregulation
Source: Front Psychiatry. 2016 Aug 24;7:146. doi: 10.3389/fpsyt.2016.00146 (PMC4995201; doi:10.3389/fpsyt.2016.00146)
Supplement: Supplementary file 1 [file Table_1.DOCX]

| **Supplemental Table 1. Items Included in the CBCL-Mania Scale, CBCL-Externalizing Scale and CBCL- Pediatric Bipolar Disorder Profile** | | | | |
| --- | --- | --- | --- | --- |
| **CBCL-Subscale** | **CBCL-Items** | **CBCL-Mania Scale** | **CBCL-Externalizing Scale** | **CBCL- Pediatric Bipolar Disorder Profile** |
| **Aggressive Behavior** | 3. Argues a lot | Item Not Included | Item Included | Item Included |
|  | 16. Cruelty, bullying or meanness to others | Item Not Included | Item Included | Item Included |
|  | 19. Demands a lot of attention | Item Not Included | Item Included | Item Included |
|  | 20. Destroys his/her own things | Item Not Included | Item Included | Item Included |
|  | 21. Destroys things belonging to his/her family or others | Item Not Included | Item Included | Item Included |
|  | 22. Disobedient at home | Item Not Included | Item Included | Item Included |
|  | 23.Disobedient at school | Item Not Included | Item Included | Item Included |
|  | 37. Gets in many fights | Item Included | Item Included | Item Included |
|  | 57. Physically attacks people | Item Not Included | Item Included | Item Included |
|  | 68. Screams a lot | Item Not Included | Item Included | Item Included |
|  | 86. Stubborn, sullen, or irritable | Item Not Included | Item Included | Item Included |
|  | 87. Sudden changes in mood or feelings | Item Included | Item Included | Item Included |
|  | 88. Sulks a lot | Item Not Included | Item Included | Item Included |
|  | 89. Suspicious | Item Included | Item Included | Item Included |
|  | 94. Teases a lot | Item Included | Item Included | Item Included |
|  | 95. Temper tantrums or hot temper | Item Not Included | Item Included | Item Included |
|  | 97. Threatens people | Item Not Included | Item Included | Item Included |
|  | 104. Unusually loud | Item Included | Item Included | Item Included |
| **Delinquent Behavior**  **(Rule-breaking behaviour)** | 2. Drinks alcohol without parent’s approval | Item Not Included | Item Included | Item Not Included |
|  | 26. Doesn’t seem to feel guilty after misbehaving | Item Not Included | Item Included | Item Not Included |
|  | 28. Breaks rules at home, school, or elsewhere | Item Not Included | Item Included | Item Not Included |
|  | 39. Hangs with others who get in trouble | Item Not Included | Item Included | Item Not Included |
|  | 43. Lying or cheating | Item Not Included | Item Included | Item Not Included |
|  | 63. Prefers being with older kids | Item Not Included | Item Included | Item Not Included |
|  | 67. Runs away from work | Item Not Included | Item Included | Item Not Included |
|  | 72. Sets fires | Item Not Included | Item Included | Item Not Included |
|  | 73. Sexual problems | Item Not Included | Item Included | Item Not Included |
|  | 81. Steals at home | Item Not Included | Item Included | Item Not Included |
|  | 82. Steals outside the home | Item Not Included | Item Included | Item Not Included |
|  | 90. Swearing or obscene language | Item Not Included | Item Included | Item Not Included |
|  | 96. Thins about sex too much | Item Included | Item Included | Item Not Included |
|  | 99. Smokes, chews, or sniffs tobacco | Item Not Included | Item Included | Item Not Included |
|  | 101. Truancy or unexplained absence | Item Not Included | Item Included | Item Not Included |
|  | 105. Uses dugs/alcohol for nonmedical purposes | Item Not Included | Item Included | Item Not Included |
|  | 106. Vandalism | Item Not Included | Item Included | Item Not Included |
| **Attention Problems** | 1.Acts too young for his/her age | Item Not Included | Item Not Included | Item Included |
|  | 4. Fails to finish things he/she starts | Item Not Included | Item Not Included | Item Included |
|  | 8. Can’t concentrate, can’t pay attention for long | Item Not Included | Item Not Included | Item Included |
|  | 10. Can’t sit still, restless or hyperactive | Item Included | Item Not Included | Item Included |
|  | 13. Confused or seems to be in a fog | Item Not Included | Item Not Included | Item Included |
|  | 17. Daydreams or gets lost in his/her thoughts | Item Not Included | Item Not Included | Item Included |
|  | 41.Impulsive or acts without thinking | Item Included | Item Not Included | Item Included |
|  | 61. Poor school work | Item Not Included | Item Not Included | Item Included |
|  | 78. Inattentive or easily distracted | Item Included | Item Not Included | Item Included |
|  | 80. Stares blankly | Item Not Included | Item Not Included | Item Included |
| **Anxious/Depressed** | 14. Cries a lot | Item Not Included | Item Not Included | Item Included |
|  | 29. Fears certain animals, situations or places other than school | Item Not Included | Item Not Included | Item Included |
|  | 30. Fears going to school | Item Not Included | Item Not Included | Item Included |
|  | 31. Fears he/she might think or do something bad | Item Not Included | Item Not Included | Item Included |
|  | 32. Feels he/she must be perfect | Item Not Included | Item Not Included | Item Included |
|  | 33. Feels or complains that no one loves him/her | Item Not Included | Item Not Included | Item Included |
|  | 35. Fells worthless or inferior | Item Not Included | Item Not Included | Item Included |
|  | 45. Nervous, high-strung, or tense | Item Not Included | Item Not Included | Item Included |
|  | 50. Too fearful or anxious | Item Not Included | Item Not Included | Item Included |
|  | 52. Feels too guilty | Item Not Included | Item Not Included | Item Included |
|  | 71. Self-conscious or easily embarrassed | Item Not Included | Item Not Included | Item Included |
|  | 91. Talks about killing self | Item Not Included | Item Not Included | Item Included |
|  | 112. Worries | Item Not Included | Item Not Included | Item Included |
| **Thought Problems** | 40. Hears sound or voices that aren’t there | Item Included | Item Not Included | Item Not Included |
|  | 59. Plays with own sex parts in public | Item Included | Item Not Included | Item Not Included |
|  | 60. Plays with own sex parts too much | Item Included | Item Not Included | Item Not Included |
|  | 70. Sees things that aren’t there | Item Included | Item Not Included | Item Not Included |
|  | 76. Sleeps less than most kids | Item Included | Item Not Included | Item Not Included |
|  | 85. Strange ideas | Item Included | Item Not Included | Item Not Included |
|  | 100. Trouble sleeping | Item Included | Item Not Included | Item Not Included |
| **Other Problems** | 74. Showing off or clowning | Item Included | Item Not Included | Item Not Included |
|  | 93. Talks too much | Item Included | Item Not Included | Item Not Included |
| **Social Problems** | 34. Feels others are out to get him/her | Item Included | Item Not Included | Item Not Included |
| CBCL=Child Behavior Checklist; Item numbers refer to the CBCL 6-18; Shaded cells indicate overlapping items across all three scales | | | | |
